# Biochar Impacts on Soil Silicon Dissolution Kinetics and their Interaction Mechanisms

**DOI:** 10.1038/s41598-018-26396-3

**Published:** 2018-05-23

**Authors:** Yaofeng Wang, Xin Xiao, Baoliang Chen

**Affiliations:** 1 Department of Environmental Science, Zhejiang University, Hangzhou, Zhejiang, 310058 China; 2 Zhejiang Provincial Key Laboratory of Organic Pollution Process and Control, Hangzhou, Zhejiang, 310058 China

## Abstract

Effects of biochars on soil silicon dissolution kinetics remain unaddressed. Si-rich rice husk (RH) and rice straw (RS), and Si-deficient wood sawdust (WB) and orange peel (OP) were applied to prepare biochars at 300–700 °C. The silicon dissolution of Si-rich biochars was relatively high in comparison with Si-deficient biochars, and increased with the pyrolysis temperature. The mechanism of silicon release is suggested to be controlled by a protective carbon-silicon interaction, as accompanied by carbon release. After mixing with soil, the addition of Si-rich biochar leads up to 72.7–121% improvement in silicon dissolution in a high-silicon soil (HSS) compared to 147–243% improvement in a low-silicon soil (LSS). The total cumulative amount of silicon dissolved decreased compared to the theoretical value due to the adsorption of silicic acid by the biochar. The addition of WB700 or OP700 as Si-deficient biochars leads to a cumulative Si dissolution decrease of 15.7 and 12.1%, respectively. The adsorption of silicic acid in the biochar and the protection of soil dissolved Fe make biochar a reservoir of soil silicon. Thus, Si-rich biochar could serve as a source of Si with slow release, while Si-deficient biochar could serve as an extra Si sink in agricultural paddy soil.

## Introduction

Biochar is a carbon-rich solid produced by the pyrolyzation of biomass (feedstock) at a relative low temperature (100 to 700 °C) under anoxic conditions^1,2^. Many studies have focused on biochar’s organic component because of its function in carbon sequestration^3–7^ and environmental remediation such as the adsorption of contaminants by oxygen-containing functional groups^1,8–11^. However, the function and effects of biochar on the surface of an inorganic component have not been fully understood, especially for biochars that contain more minerals. Silicon is the second most abundant element in the earth’s crust^12^, and its content in some silicophilic plant-derived biochars such as rice straw could reach around 20% (dry matter)^13^. Qian and Chen reported the phenomena for the co-precipitation of Al with silicate particles from manure biochar, which could effectively alleviate aluminum phytotoxicity in wheat^14^. Furthermore, the Si released from Al-containing biochars participated in an alumino-silicate reconstruction, resulting in the long-term alleviation of aluminum phytotoxicity in acidic soils^15^. Additionally, silicon together with carbon in biochar plays a vital role in biochar stability, affected by the aromaticity and possible protection by silicon encapsulation, and the morphology of the carbon forms, which were also greatly associated with silica. A coupled Si-C framework has been demonstrated to govern biochar carbon sequestration^3^. The silicon speciation was changed from amorphous to crystalline phase thus regulating the morphology, because of the transformation and dissolution in rice straw derived biochar. However, the organic carbon changed from aliphatic to aromatic, and the mutual protection between carbon and silicon from dissolution was proposed to account for the structure of the biochar^13^. These results indicated that silicon dissolution has a critical effect on the ability to sequester carbon. Therefore, the effect of the biochar on the silicon dissolution kinetics and equilibration in soil should be addressed to elucidate the potential agricultural and environmental applications of the biochar amendment.

After addition to the soil, biochar could act as a nutrient source because of the release of nutrients such as N, P, and K contained in the biochar^16^. Furthermore, the biogeochemical cycling of carbon and nitrogen can be impacted by biochar^17^. However, the impact of the biochar has not raised concern about the biogeochemical cycling of Si, and the effect of the biochar with different Si contents on the soil silicon equilibration is extremely important. Si is an important nutritional element for plant growth, and silicon in plant tissue defends biotic and abiotic stress such as toxic metal stress, leaf microbial pathogen resistance, and drought tolerance^18–22^. Rice is considered a silicon-rich plant that can take up silicon from the soil as soluble silicic acid^23,24^, and rice shoots contain up to 10% Si in their dry matter, and 270 kg/ha/year for the entire rice plant^25^. Rice straw and rice husks are rich sources of silicon, so carbonized straw or husk is a good silicon fertilizer^26^. *Miscanthus* biochar acted as potential bio-available Si sources^27^, and the reason was attributed to the substantially higher amount of phytolithic Si derived from grass-derived biochar than other plant-derived biochars^28^. Moreover, the effect of wheat-straw biochar on soil silicon in different regions was investigated, but the results were contradictory about whether the plant-available Si content of soil increased or decreased after biochar amendment of paddy soil, and their interaction mechanism was not investigated^29^. Nonetheless due to the continuous removal of silicon from the soil via plant uptake, the soil may lack plant-available silicon if the straw (wheat or rice) is not returned to the field. More importantly, along with Si and Al complexes from soil, an increase in Fe and soil organic matter concurred^30^. Silicic acid dissolved in the soil solution can be adsorbed to soil minerals, particularly to Fe and Al oxides/hydroxides^31–33^. Knowledge about the release of silicon from Si-rich and Si-deficient biochars under different pyrolytic temperatures is poor, and the effects of the biochar with different Si contents on soil silicon dissolution kinetics and their interaction mechanisms are unavailable.

The primary objective of this study was to demonstrate the distinct release behaviors of silicon from Si-rich and Si-deficient biochars produced under different pyrolytic temperatures and their different impacts on soil silicon equilibration. The silicon dissolution kinetics and cumulative amount of silicon dissolved from Si-rich and Si-deficient biochars were evaluated by various extraction methods. Scanning electron microscopy with energy X-ray dispersive spectrometry (SEM-EDS) was used to illustrate surface changes of the biochars before and after silicon release. An incubation experiment was carried out to evaluate the effect of biochar on soil silicon equilibration after biochars were incorporated into soils with different silicon contents. This study will improve our knowledge about the silicon equilibrium in a soil-biochar system and promote the application of the biochar as a silicon-releasing agent in agriculture to increase plant-available silicon in soils and to improve crop health.

## Results

### Structural Characteristics of Si-rich and Si-deficient Biochars

The yield, elemental composition and the surface area (SA) of the Si-rich and Si-deficient biochars are shown in Table 1. The carbon, silicon, ash content, and BET-N_2_ surface area increased with an increase in pyrolytic temperature^13^. And the biochar yield decreased. Obviously, biochar derived from RH and RS had a higher Si content (the Si-rich biochars), for example, 16.11–25.70% Si for the RH-biochars and 11.02–18.46% Si for the RS-biochars, while the biochar derived from WB and OP had a lower Si content (the Si-deficient biochars), for example, 0.30–0.37% Si for the WB-biochars and 0.30–0.43% Si for the OP-biochars. In general, silicon was the major component of the ash^13^.

**Table 1:** Yield, elemental composition, ash content, and BET-N_2_ surface area (SA) of the biochar derived from rice husks (RH), rice straw (RS), wood sawdust (WB) and orange peel (OP) at different pyrolytic temperatures. The suffix number in the biochar name indicates the carbonization temperature.

Style	Sample	Yield (%)	Ash (%)	C (%)	SiO_2_ (%)	SA (m^2^/g)
Si-rich biochars	RH300	47.65	31.22	35.47	16.11	7.921
	RH500	35.09	42.84	38.71	22.26	110.7
	RH700	30.51	50.99	46.31	25.70	226.8
	RS300	46.99	27.08	41.39	11.02	3.669
	RS500	32.15	41.45	44.96	14.46	91.99
	RS700	26.00	51.13	47.63	18.46	223.6
Si-deficient biochars	WB300	44.61	0.5850	64.98	0.2985	14.81
	WB500	20.79	1.513	70.86	0.3238	433.1
	WB700	13.32	2.144	79.89	0.3690	529.8
	OP300	37.97	5.925	59.19	0.3001	11.91
	OP500	22.83	9.145	61.93	0.3539	20.17
	OP700	15.52	10.13	72.13	0.4292	130.2

**Figure 1:**
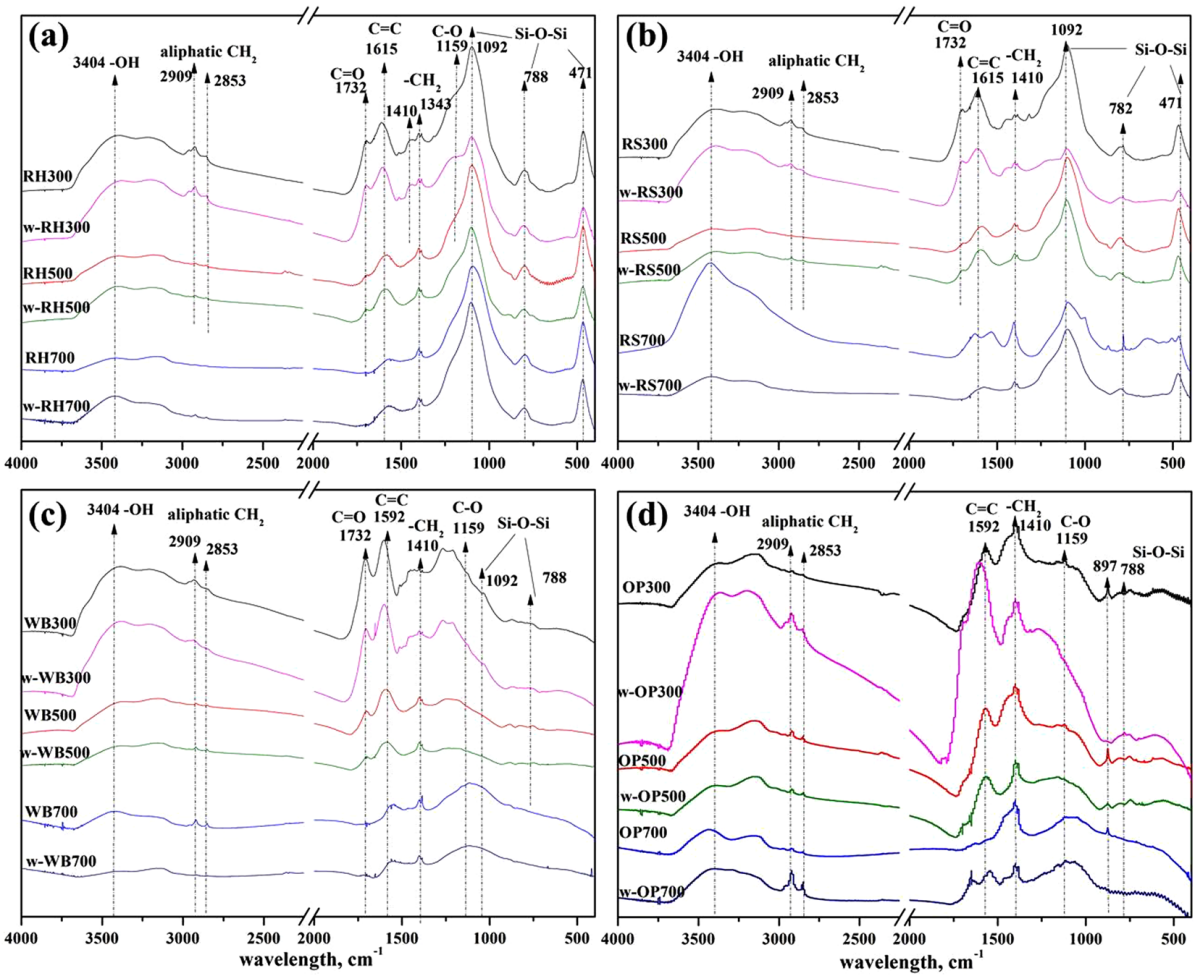
FTIR spectra of rice hull-derived biochar (RH300, RH500, and RH700) (**a**), rice straw-derived biochar (RS300, RS500, and RS700) (**b**), wood sawdust-derived biochar (WB300, WB500, and WB700) (**c**), and orange peel-derived biochar (OP300, OP500, and OP700) (**d**) before and after washing (after washing indicated with “w−”). The suffix number in the biochar name indicates the carbonization temperature.

The FTIR spectra of Si-rich and Si-deficient biochars are shown in Fig. 1. The band at 3404 cm^−1^ indicates the O–H stretching vibration. The peaks at 2909 cm^−1^, 2853 cm^−1^, 1410 cm^−1^, and 1343 cm^−1^ were assigned to –CH_2_ groups. The band at 1732 cm^−1^ represents C=O stretching, and the bands at 1615 cm^−1^ and 1592 cm^−1^ were assigned to C=C stretching vibration of the biochar^34^. The band at 1159 cm^−1^ represents the C−O of the biochar^34^. For the RH and RS biochars, the peaks at 1092 cm^−1^, 782 cm^−1^, and 471 cm^−1^ were assigned to the Si-O-Si groups^13^. In comparison, WB and OP biochars confirmed the low Si content since there was no band observed at 471 cm^−1^, which is commonly attributed to Si content. The aromaticity of biochars was found to be dominant at a higher temperature with less aliphatic content. The condensation crystallization of carbon during the charring process reduces the amount of carbon released from the biochar^13^. Similarly, the symmetrical and asymmetrical peaks at 1092 cm^−1^ represent amorphous and crystalline silicon, respectively^35^, and the Si morphology of the RS700 and RH700 biochars can be changed from amorphous to crystalline during the carbonization process (Figure S1).

**Figure 2:**
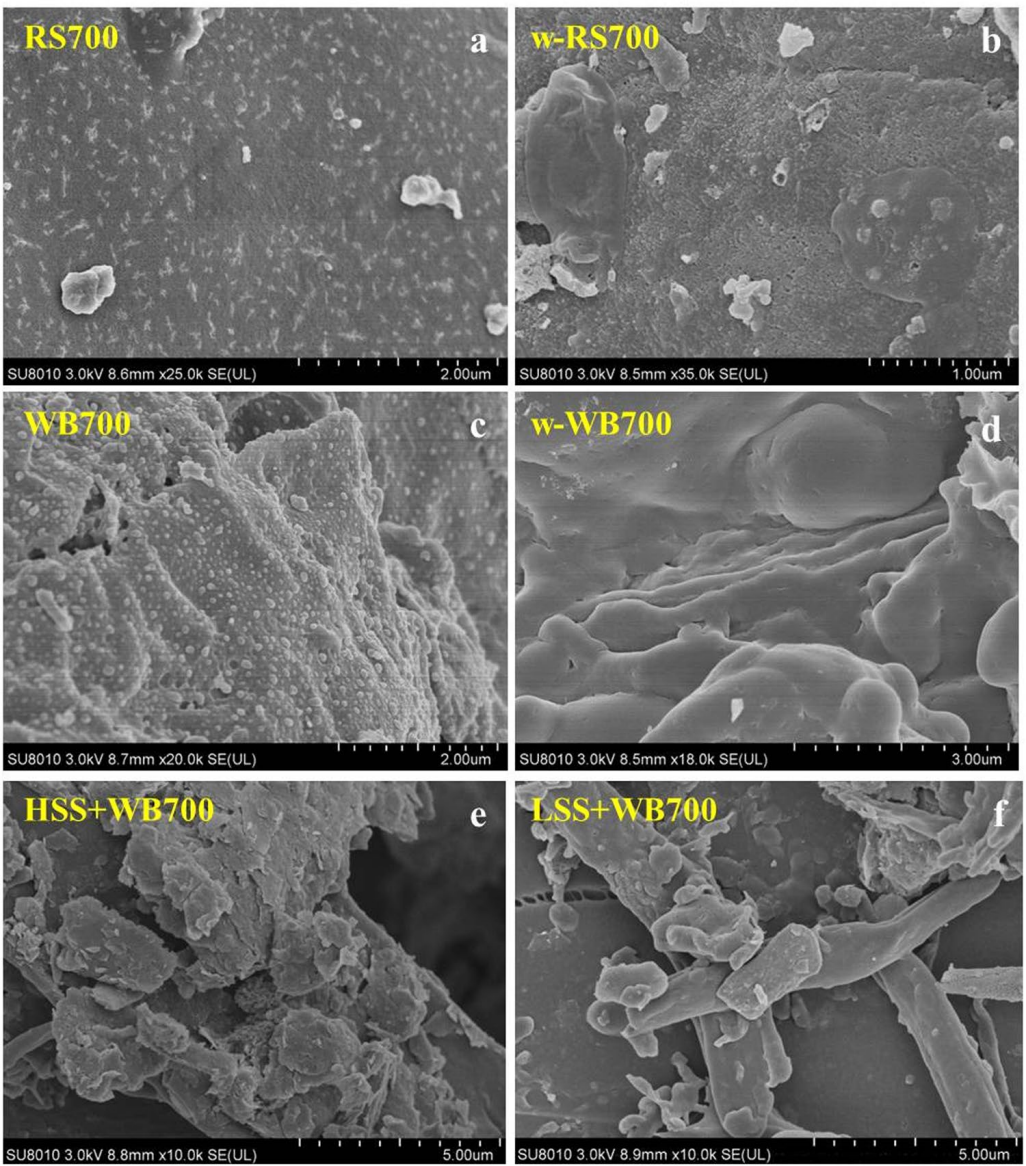
SEM images of the RS500 (**a**,**b**) and OP500 (**c**,**d**) before and after washing, biochar and soil (HSS and LSS) mixtures (**e**,**f**). The suffix number in the biochar name indicates the carbonization temperature.

SEM images of the biochars and biochar-soil mixtures are shown in Fig. 2. Figure 2a shows white, regularly shaped substances, which are silicon dioxide sediments (phytoliths) that were found on the internal surface of the RS500 biochar^36^. However, the small white pellet on the surface of OP500 biochar may be carbon sediment (Fig. 2c). Precipitation of minerals on the surface of biochars was not obvious (Fig. 2a,c). On the contrary, the mineral contents were found on the surface of biochar when the biochar was mixed with soil (Fig. 2e,f). It would be useful to understand how the biochars change the Si-containing minerals. After all, this is part of the interaction that is the focus of the paper.

### Silicon Dissolution Characteristics of the Si-rich and Si-deficient Biochars

**Figure 3:**
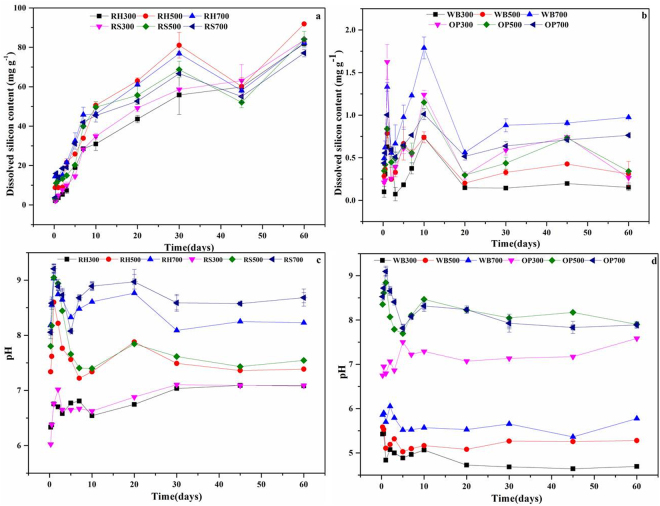
Silicon dissolution kinetics of the RH and RS biochars (**a**), the WB and OP biochars (**b**) and the corresponding pH variations in the RH and RS biochars (**c**) and WB and OP biochars (**d**). The samples include the Si-rich biochars RH300-RH700, RS300-RS700 and the Si-deficient biochars WB300-WB700, OP300-OP700. The solid-to-liquid ratio is 50 mg/50 mL. The suffix number in the biochar name indicates the carbonization temperature. Note that the maximum.

Jones and Handreck designated two categories related to the amount of Si available in biomass materials^37^, Si-rich (i.e., RH, RS) and Si-deficient (i.e., WB, OP). The silicon dissolution kinetics of the Si-rich biochars and the Si-deficient biochars are shown in Fig. 3a,b, respectively, and the corresponding pH variations are presented in Fig. 3c,d, respectively. During the first 12 h, the pH values peaked (Fig. 3c), leading to rapid silicon dissolution rate (for example the peak value of RS700 was approximately 10.0). After that, the rate of silicon dissolution gradually decreased, and the dissolved silicon content after 45 d was lower than that at 30 d or 60 d. For the Si-deficient biochars, silicon was released rapidly during the first 12 h, and the higher silicon dissolution rate was related to the relatively higher pH (Fig. 3d). The rate fluctuated from 12 h to the first 10 d, probably because the molecules of silicon acid may be absorbed on the surface of biochar. Although the silicon release for the Si-deficient biochars showed less dissolved silicon after 10 d, the dissolved silicon content for the Si-deficient biochars became stable afterward, possibly because of the silicon equilibrium between the biochar surface and the solution after 20 d. In other words, an equilibrium between adsorbed silicon and silicon dissolved from biochar surface was established.

**Figure 4:**
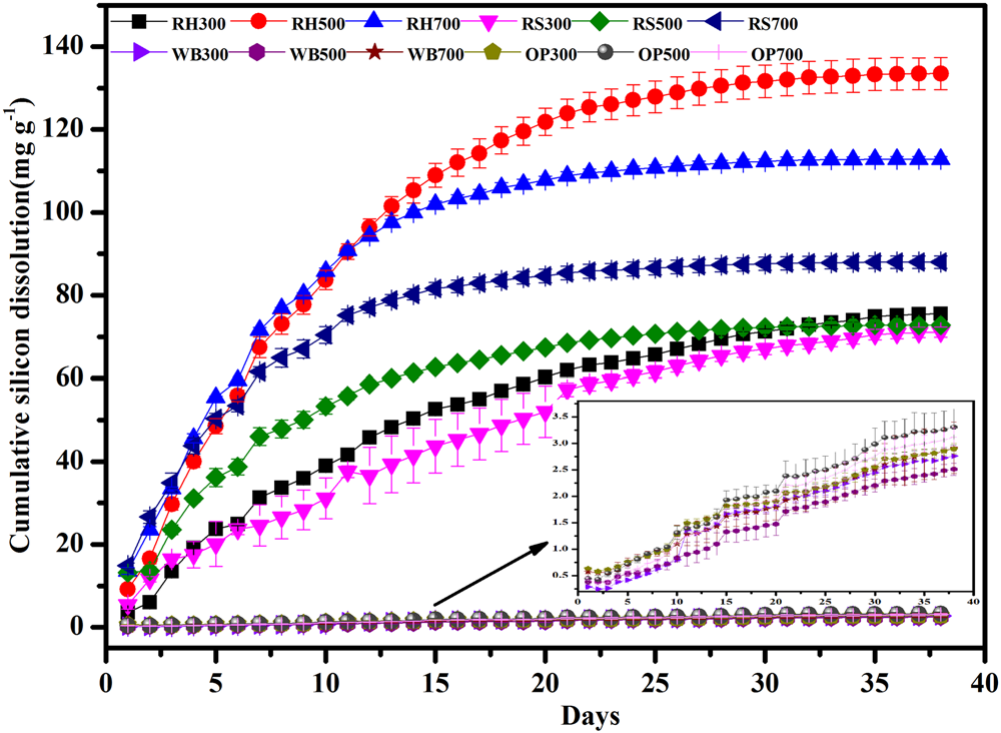
Cumulative amount of silicon dissolved for the RH and RS biochars (RH300, RH500, and RH700; RS300, RS500, and RS700) and the WB and OP biochars (WB300, WB500, and WB700; OP300, OP500, and OP700). The solid-to-liquid ratio was 50 mg/50 mL. The suffix number in the biochar name indicates the carbonization temperature.

Repeated extraction of silicon from the biochars with fresh solution was carried out and are presented in Fig. 4. The final cumulative silicon release curves for the Si-rich (i.e., RH and RS) biochars and Si-deficient (i.e., WB and OP) biochars were obtained after the 38-day extraction. An extraction duration of 38 days was greater than the 20 days reported in a previous study^13^, which may be related to the precursory material of the biochars. Figure 4 shows that the Si-rich (i.e., RH and RS) biochars have higher final cumulative amount of silicon dissolved. The trend for the Si-rich (i.e., RH and RS) biochars shows a relatively higher silicon dissolution rate for the first 10 days and then a gradual decrease to a lower rate before a silicon release rate of approximately zero is finally reached. The reason for this trend can be attributed to the high content of silicon in the biochar (Table 1). The dissolution rates of the cumulative silicon release from Si-rich biochars (i.e., RH and RS) at different pyrolytic temperatures are almost the same, but the total Si release from the RH500 biochar is greater than that for the other RH biochars, consistent with the results of Xiao^13^. On the other hand, the Si-deficient (i.e., WB and OP) biochars showed a relatively low final cumulative amount of silicon dissolved, and the rate of silicon release consisted of two stages, a higher rate stage and a lower rate stage. The Si-deficient biochars showed no differences with different pyrolysis temperatures due to the low Si release (Table 1).

### Effect of Biochar on Soil Silicon Dissolution

**Figure 5:**
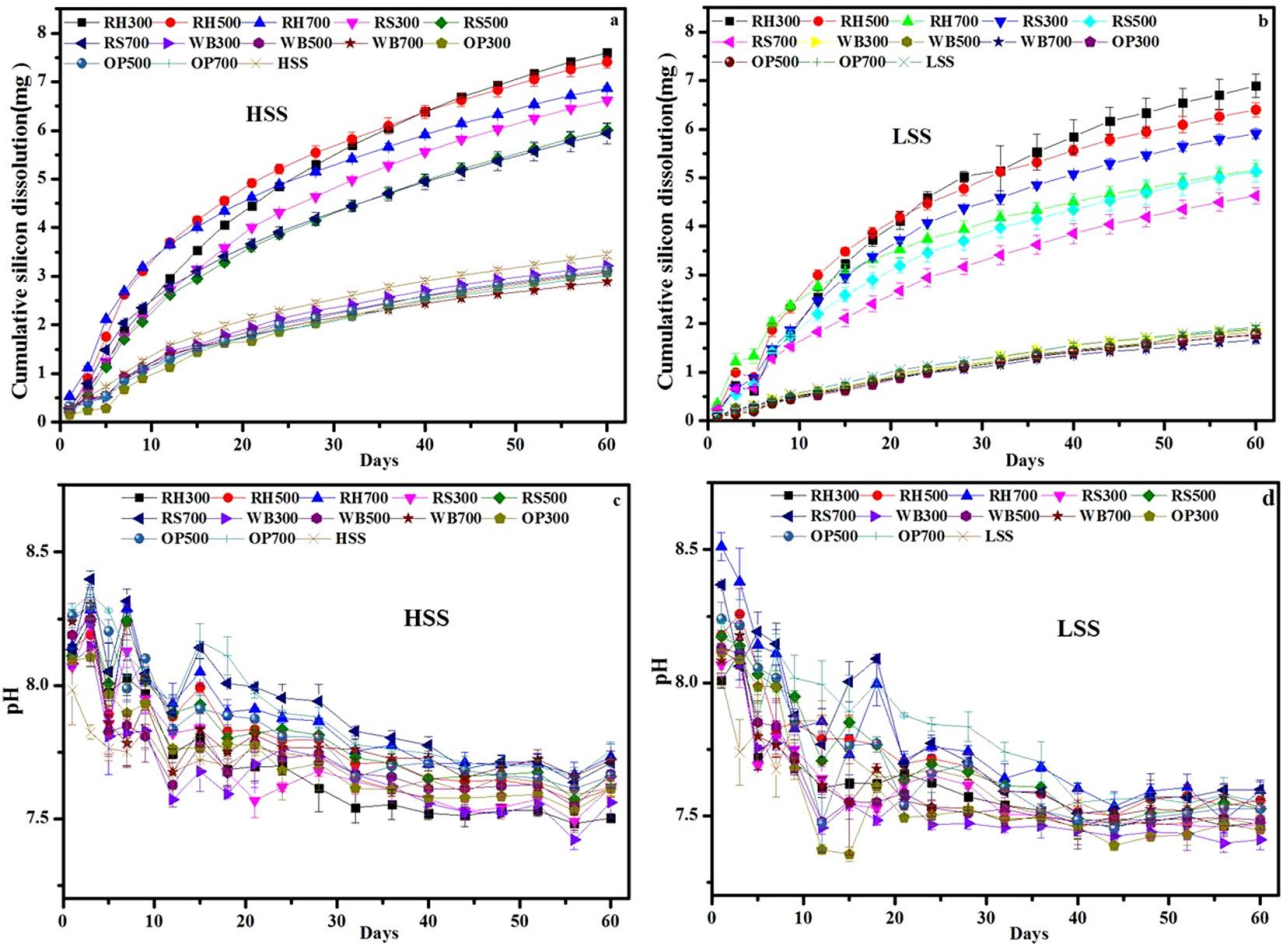
Cumulative amount of silicon dissolved of high-availability silicon soil + biochars (**a**), and low-availability silicon soil + biochars (**b**) and corresponding daily pH values (**c**,**d**).The solid-to-liquid ratio was 50 mg biochar 2000 mg soil/50 mL. The suffix number in the biochar name indicates the carbonization temperature.

**Figure 6:**
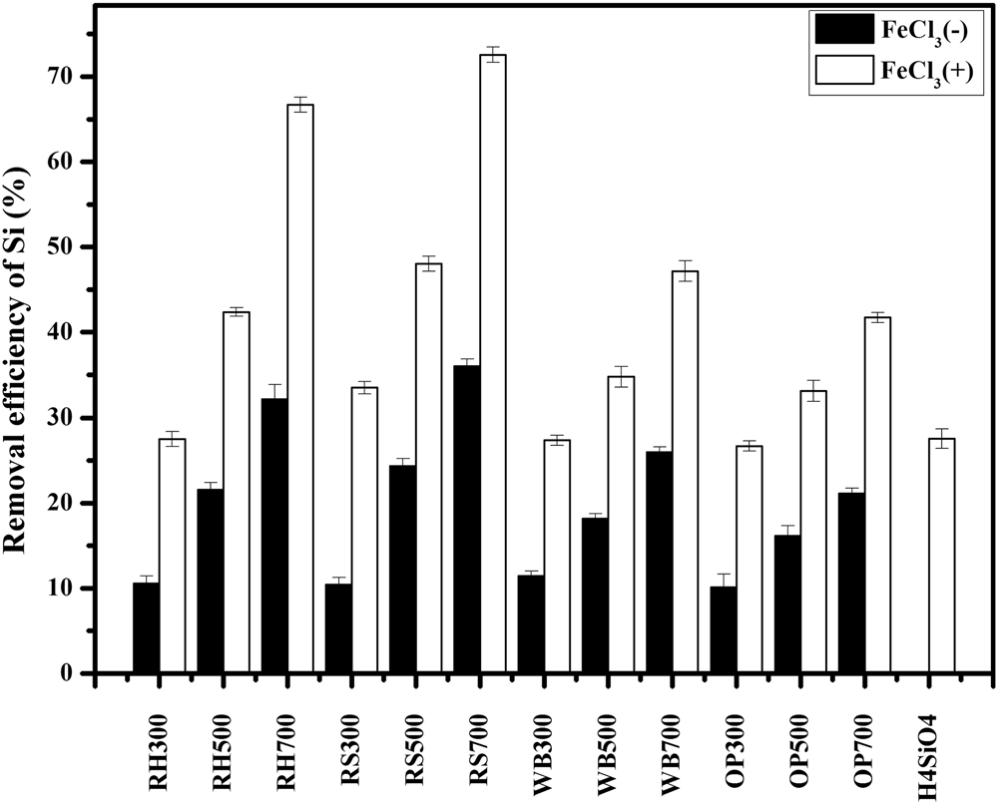
Removal efficiency of the biochars on the silicic acid (6 mg/L) with or without FeCl_3_. The samples included the Si-rich biochars (RH300-RH700, RS300-RS700) and low-availability silicon mixtures Si-deficient biochars (WB300-WB700, OP300-OP700). The solid-to-liquid ratio is 50 mg/10 mL. The suffix number in the biochar name indicates the carbonization temperature.

Figure 5a,b show the soil final cumulative Si dissolution curves of HSS and LSS, respectively, mixed with Si-rich and Si-deficient biochars. Figure 5a shows that, compared to HSS, the final cumulative soil silicon dissolution amount with Si-rich biochars was increased by approximately 72.7% to 121%. Although the amount of silicon released from a pure Si-rich biochar increased with the pyrolysis temperature, the degree of increase slightly decreased with the pyrolysis temperature, which may be related to the balance between the re-adsorption of silicic acid by the pores of the biochar carbon structure (Fig. 6) and the silicon released from the silicon component of the biochar. Similarly, in a low-silicon soil, the final cumulative soil silicon dissolution amount of Si-rich biochar was increased by 147% to 243%, and the degree of increase decreased with the pyrolysis temperature (Fig. 5b). The improvement in soil silicon dissolution incorporated with Si-rich biochars was not correlated with soil available silicon content. And this increasing trend of the Si-rich biochar for LSS was better than that for HSS, possibly because of the high silicon dissolution for Si-rich biochars (Figs 3a and 4a), and the low soil available content in LSS and the increased pH in the first 7 days (Fig. 5c,d). Thus, a portion of the Si released from the Si-rich biochars can be integrated into the soil silicon to augment the soil available silicon. The release of Si from the WB700 biochar was decreased by 15.7%, and it was decreased by 12.3% from the OP700 biochar as compared to HSS as shown in Fig. 5a. The addition of WB700 decreased the Si release by 12.1%, while the addition of the OP biochars showed no effect on the Si release compared to LSS (Fig. 5b), which was probably related to the soil available silicon content, re-adsorption of silicic acid by the biochar (Fig. 6) and binding with Fe Al minerals.

## Discussion

### Silicon Dissolution from Different Biochars

The release of silicon from Si-rich biochars and Si-deficient biochars, peaked during first 10 days, and corresponding periodical peaks are shown in Fig. 3a,b. We suggest that the silicon released by biochars may be controlled by other surface components of biochar such as C and Si^13^. However, the amount of silicon released by Si-rich biochars (approximately 100 mg g^−1^ for 60 d) is much higher than that of Si-deficient biochars (approximately 1.0 mg g^−1^ for 60 d), which means that Si-rich biochars with greater ash content exhibit higher silicon dissolution, and Si-deficient biochars with lower ash content exhibit a lower silicon dissolution. The surface of Si-rich biochars was found to be porous after silicon was release (Fig. 2b), which might correspond to the position of silica or the phytoliths. Presumably, such pores on the surface of biochar may be effective sites for silicic acid adsorption. This phenomenon primarily indicates a marked reduction in the silicon dissolution kinetics of the RH, and RS biochars after 45 d (Fig. 3a). Considerably, the portion of Si derived from Si-rich or Si-deficient biochar had a great impact on the soil silicon content.

The total organic carbon (TOC) of Si-rich biochars remained unchanged before and after washing, and TOC for Si-deficient biochars after washing was decreased remarkably (Figure S2), indicating that high silicon dissolution leads to carbon loss during washing, which could be further attributed to the protective interaction between carbon and silicon. The SEM images of biochars after washing are shown in Fig. 2. The surface of RS500 after washing had abundant pores, which may point out the position of silicon and minerals; however, the surface of OP500 was smooth with few evident pores after washing. The pores on the surface of biochar may be the major adsorption sites for silicic acid. The change in FTIR spectra at 1092 cm^−1^ indicates that the Si morphology in biochars after washing can change from crystalline form to an amorphous form during the carbonization process^35^. In other words, the Si species on biochar can be converted to amorphous silica by washing (Figure S1)^38^.

The ash in biochar is one of the major reasons for its alkalinity. The Si species released from RH- and RS biochars is mainly silicate, and thus, the rate of silicon dissolution at pH > 7 is relatively higher during first seven days, then no further pH effect is evident for RS biochars. The reason could be attributed to the silicon release, which may be controlled by carbon-silicon structural model once the ash dissolves^13^, highlighting the great impact of Si dissolution on the ash content of biochar. The cumulative silicon release for high ash biochar (i.e., RH and RS) is much more than that of the low ash biochars (i.e., WB and OP). However, the efficiency of the Si dissolution is reversed (Figure S3), which is related to the ash content and characteristics of the biochar. Phytoliths account for 90% of rice straw silicon^26^, and because of the phytoliths Si, RH- and RS-biochars have been designated phytolithic biochars, while WB- and OP-biochars are called non-phytolithic biochars. The first stage is dissolution of non-phytolithic silica at alkaline pH and attack of the Si-O-Si bond by hydroxyl ions, and the second stage is dissolution of phytoliths (RH, RS biochars). Phytoliths, which are highly resistant to dissolution under natural physicochemical conditions, can remain in the soil for thousands of years^39^. However, recent studies report that phytoliths consists of SiO_2_ (66–91%), organic carbon (1–6%), Fe (0–2.1%), and Al (0.01–4.55%)^40^, and the solubility of phytoliths of rice cultivars is controlled by the chemical composition, morphology (i.e., the specific surface area) and the hydration rate^41^. Therefore, the rate of Si dissolution with Si-rich (phytoliths) biochars is less than that for Si-deficient (non-phytoliths) biochars. In addition, although the pH for the OP biochar solution had relatively greater change (for OP700, the pH changed from 9.3 to 6.5) (Fig. 5d), the amount of silicon released was small. Moreover, the pH of WB300 and WB500 was below 7 due to the low ash content in biochar (Fig. 5c), and the silicon released made no difference during first ten days.

### Si Balance Derived from Soil and Biochars

**Figure 7:**
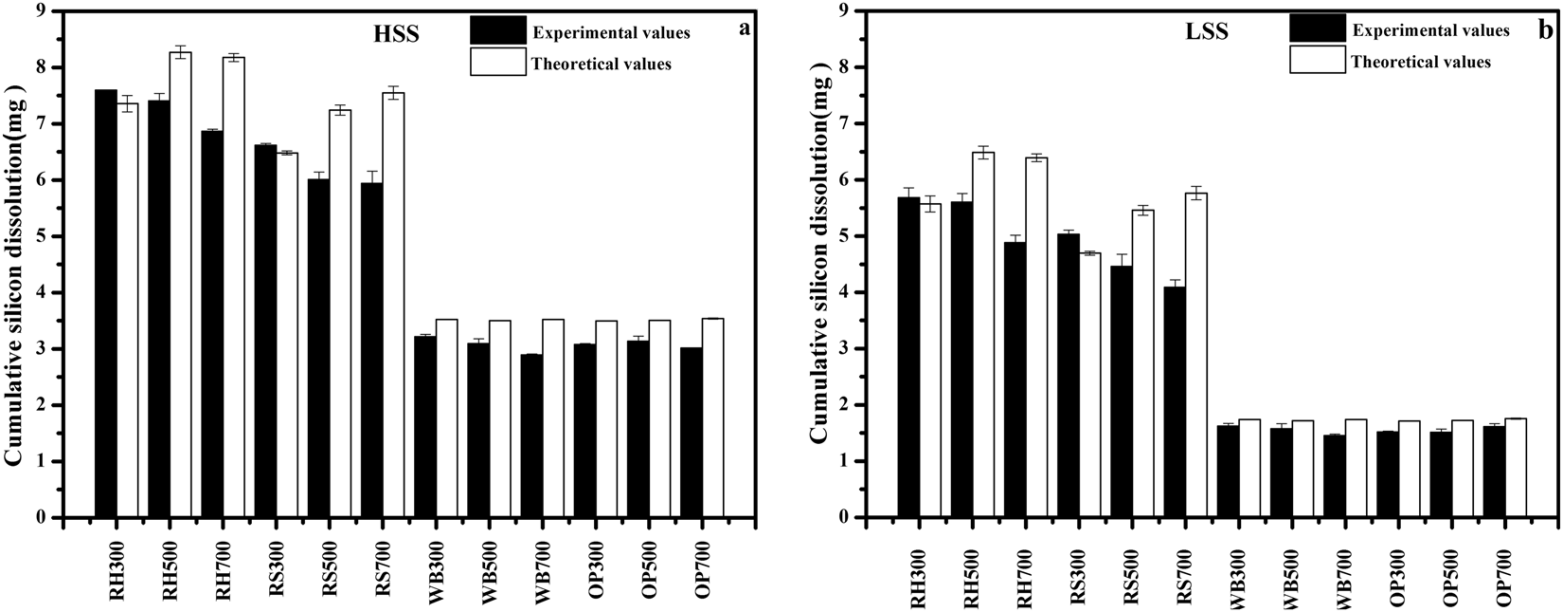
Cumulative amount of silicon dissolved from 50 mg biochar + 2000 mg soil tubes of (**a**) highly available silicon soil (HSS) and (**b**) low-availability silicon soil (LSS). Experimental values are obtained from the experiment of biochar-soil mixtures (biochar amended soil), which are the determined data of cumulative amount of silicon dissolved in the system of the biochar-soil mixtures. And theoretical values are calculated via the sum of cumulative amount of silicon dissolved from biochars and soil (HSS and LSS) individually. The suffix number in the biochar name indicates the carbonization temperature.

**Figure 8:**
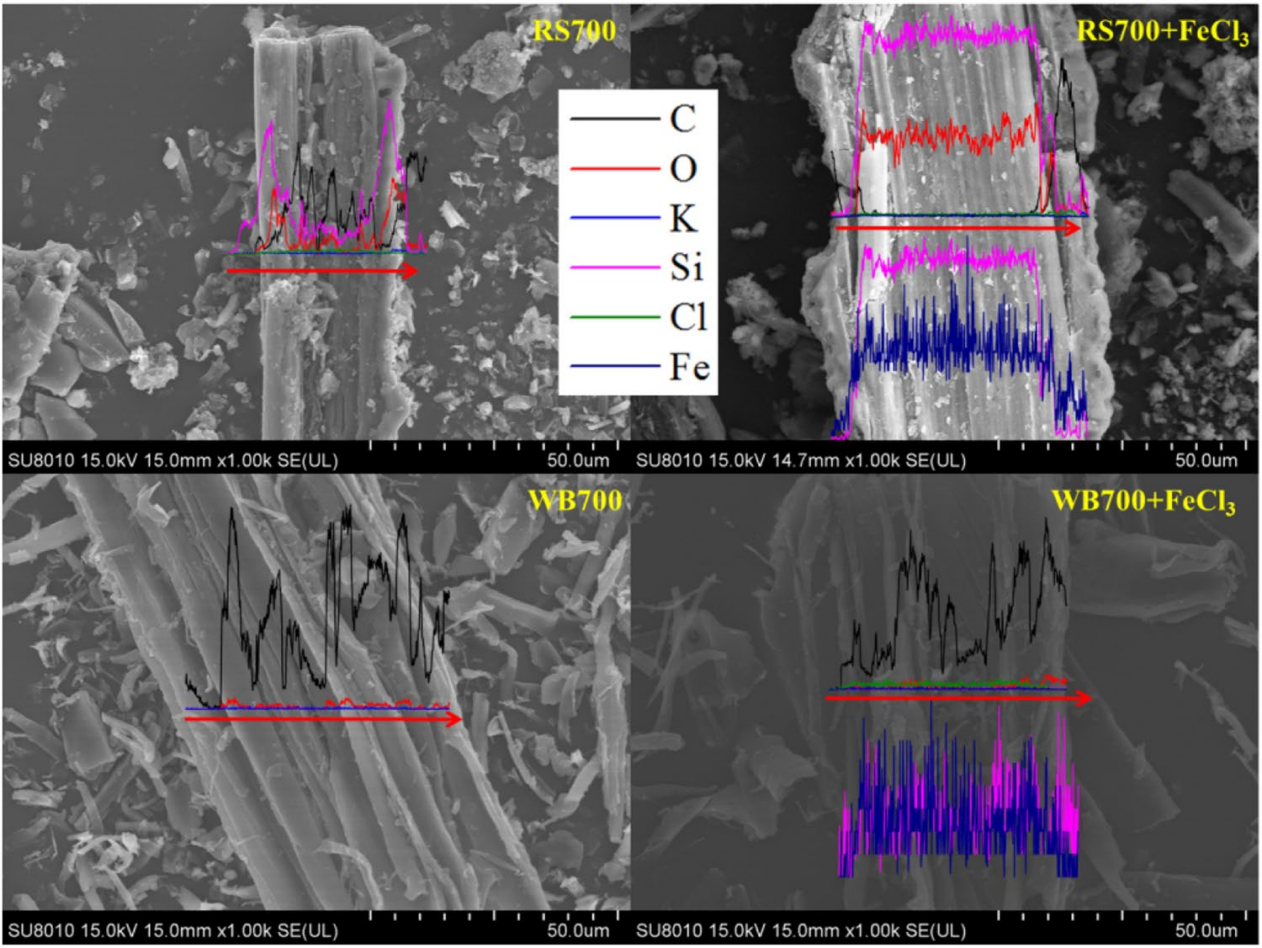
SEM-EDS images of RS700 and WB700 after the absorption of silicic acid with or without FeCl_3_. Black line, red line, blue line, magenta line, olive line, and navy line refer to C, O, K, Si, Cl, and Fe, respectively. The suffix number in the biochar name indicates the carbonization temperature.

It has been known that Si-rich and Si-deficient biochars respectively have a high or low silicon release rate. The amount of silicon dissolved will change if Si-rich or Si-deficient biochar is incorporated into soil with different silicon content. Figure 7 shows the theoretical values for the cumulative amount of silicon dissolution and the experimental values for different biochar-soil mixtures. For Si-rich biochars (i.e., the RH and RS biochars), the higher improvement in silicon dissolution in a high-silicon soil (HSS) compared to that in a low-silicon soil (LSS) treated with Si-rich biochars was observed. On the one hand, the Si-rich biochars was a higher silicon dissolution and it can improve the soil available silicon in the HSS and LSS. On the other hand, the difference between the soil available Si of HSS and LSS led to the difference of improvement percentage. The cumulative amount of silicon dissolution derived from the biochar-soil mixtures was below the theoretical value, except for RH300 and RS300, which was calculated from the summary of individual biochars and soils, and this phenomenon was not affected by the soil silicon content. We suggest that the interaction between the soil and the biochar may have caused the change, but it is not certain whether the amount of soil silicon released was less or less silicon dissolved from the biochar in the biochar-soil mixture. One possible reason is that the silicic acid in solution was sorbed by the original biochar or by the biochar after washing. The sorption process is illustrated in Fig. 6 FeCl_3_(−). Another reason is that the solution of Si and iron are fixed on the biochar surface (Fig. 6 FeCl_3_(+)). Previous studies indicate that Si can readily adsorb onto mineral surfaces^31–33^, and reports indicate the possible adsorption of iron or iron compounds on the surface of the aged biochar^42^. Figure 6 shows that silicic acid in solution can be removed by adding FeCl_3_, and thus, the removal percentage of silicic acid along with ferric iron by Si-rich biochars is significantly greater than for silicic acid alone, and it increases with the surface area of the biochar. We suggest that the increased removal efficiency is related to the iron adsorbed to the biochar. The line scanning EDS spectra in Fig. 8 shows that the distribution of Si and Fe elements are consistent. For Si-deficient biochars (i.e., the WB and OP biochars), the cumulative amount of silicon dissolved derived from the biochar-soil mixture was less than the theoretical value (biochar alone plus soil (HSS or LSS) alone) (Fig. 7), and it is greater for Si-rich than Si-deficient biochar. Similarly, the removal efficiency with added ferric iron is better than that for silicic acid alone, and the iron precipitation was indicated on the biochar surface by SEM-EDS, and the content of silicon in the treatment of WB700 with FeCl_3_ is similar to that of iron (Fig. 8). As a result of the low Si release for the Si-deficient biochars, the Si removal effect with added ferric iron by WB700 is less than that by RH700 and RS700, although the WB700 has the largest surface area of all of the biochars. Regardless of the Si content of the biochar, the portion of Si derived from the soil and the biochar can finally be fixed on the biochar surface. Although the process on the biochar surface may require assistance from Fe, the biochar is considered a silicon reservoir.

### Environmental Implications

Si, which is usually coupled with carbon (C), plays a significant role in different terrestrial biogeochemical processes^43^. However, the silicon content in silicon-rich and silicon-deficient biochar has not raised concern. The Si-rich biochar could serve as a source of Si with slow release, while a Si-deficient biochar could serve as an extra Si sink in agricultural paddy soil. Studies of silicon dissolution in single biochars and biochar-soil mixtures indicate the importance of soil silicon for biochars with different silicon contents. Moreover, the effect of wheat-straw biochar (Si-rich biochar) on soil silicon in different regions was investigated, but the results were contradictory about whether the plant-available Si content of soil was increased or decreased after biochar amendment of paddy soil, and that may be related to feedstocks and pyrolysis conditions of biochar. More studies are needed to focus on the silicon biogeochemical cycles and the silicon transport distribution between terrestrial and marine ecosystems. Si-rich biochars can create a potentially biogenic silica pool^44^, and but the effect of Si-deficient biochars in the silicon geochemical cycle is uncertain. On the other hand, biochar has been applied to paddy soils as a carbon sequestering method and the rice growth consumes much silicon, and it is uncertain whether a Si-deficient biochar can be an effective soil additive for rice production. Further studies should focus on the silicon source (biochar or soil) in paddy soil. This study supports the application of the biochar as a silicon-releasing agent in agriculture to increase plant-available silicon in soils to improve crop health.

## Methods

### Soil Sampling and Preparation

Two different plant-available Si content soil samples were collected from a paddy field located in Zhejiang University, China. The mean annual temperature and rainfall are 15–18 °C and 980–2000 mm in this zone, respectively. The soil (at site 30°18′32.87″N 120°04′20.78″E) with high available Si content was derived from a paddy field (fertilizer) and was named as HSS, while the soil (at site 30°18′27.00″N 120°04′31.21″E) with low Si content was derived from backfill soil (no fertilizer) and was named as LSS. The HSS was clay loam and contained 34% clay (<0.002 mm), 34% silt (0.002–0.02 mm), and 32% sand (0.02–2 mm), whereas LSS was sandy soil with 21% clay (<0.002 mm), 16% silt (0.002–0.02 mm), and 63% sand (0.02–2 mm). The samples were air-dried, passed through a 2-mm sieve and mixed thoroughly before use. The properties such as pH (1:2.5H_2_O), available SiO_2_ content, TOC and amorphous Fe for HSS were 8.04, 120.4 mg kg^−1^, 0.801%, 3.32% and 7.89, 62.68 mg kg^−1^ 0.554%, 2.29% for LSS. The method that measures soil properties in the soil samples is listed in the Supporting Information.

### Preparation of Si-rich and Si-deficient Biochars

The parent biomass was selected based on Si content such as rice husk (RH) and rice straw (RS) for high Si-rich biochars whereas pine wood sawdust (WB) and orange peel (OP) for Si-deficient biochars for the preparation of biochars. The content of Si in the RH, RS, WB, and OP are 9.23%, 6.58%, 0.06%, and 0.05%, respectively^32^. RH and RS were obtained from an agricultural farm located in Zijingang Campus of Zhejiang University. WB was obtained from a wood factory based in Huzhou, Zhejiang, China, and OP was collected from the fruit store located in Zijingang campus, Zhejiang University. Two types of the biochars were produced at three different temperatures (300, 500 and 700 °C) as reported earlier^11^. The biochars samples were named as parent material and temperature, i.e., RH300, RH500, RH700 where the suffix number in biochar name indicates the carbonization temperature and so on (RS300, RS500, RS700, WB300, WB500, WB700, OP300, OP500 and OP700). Finally, the biochar samples were passed through a 100-mesh sieve for subsequent experiments. The method that measures biochar characteristics in the biochar samples is listed in the Supporting Information.

### Dissolution Characteristics of Silicon Dissolved from Biochars

The dissolved silicon release from biochars was evaluated by a batch continuous kinetic release and extraction over time. For the dissolution kinetics, 50 mg of biochar sample was mixed with 50 mL of 0.02 mol L^−1^, CaCl_2_ in 50-mL plastic centrifuge tube and the tube was shaken at 120 rpm, 25.0 ± 0.5 °C for 0, 0.25, 0.5, 1, 2, 3, 5, 7, 10, 20, 30, 45, and 60 d. The solution-phase Si was analyzed in the dissolution kinetic study. The samples were centrifuged at 3000 rpm for 5 min, and the concentration of dissolved silicon was examined by using Si-molybdenum blue colorimetry with detection limit 0.1 mg L^−1^ and simultaneous pH monitoring.

The potential Si release capacity from Si-rich and Si-deficient biochars at different temperatures was evaluated by repeated extractions as reported earlier^11^. Dissolved silicon was extracted using 0.02 mol L^−1^, CaCl_2_, and the samples were washed with Milli-Q water followed by vacuum drying for 12 h at 60 °C. The resulted samples were denoted as w-RH300, w-RH500, w-RH700, w-RS300, w-RS500, w-RS700, w-WB300, w-WB500, w-WB700, w-OP300, w-OP500, and w-OP700. The surface transformation of biochars after washing with water was observed by SEM. All samples were prepared in duplicate with blank samples, i.e., without biochar were also prepared. The curve or column diagram can be drawn using average values and standard error in duplicate sample by Origin 8.5 or Excel 2007 software.

### Effect of the Biochar on Soil Silicon Dissolution

To investigate the continuous release of silicon in the HSS and LSS soils, 50 mg of Si-rich or Si-deficient biochar samples was mixed with 2000 mg HSS or LSS soil in 50 mL plastic centrifuge tubes, and then 50 mL Milli-Q water was added. The soil and the biochar were well mixed, and the tubes were shaken at 25.0 ± 0.5 °C and 120 rpm for 1, 3, 5, 7, 9, 12, 15, 18, 21, 24, 28, 32, 36, 40, 44, 48, 52, 56, and 60 d. At each time, a 50 mL centrifuge tube was selected and centrifuged at 3000 rpm for 5 min. The concentration of dissolved silicon was examined by using Si-molybdenum blue colorimetry. For pH and silicon measurement, 40 mL solution was removed and replaced with 40 mL of distilled water. The soil silicon repeated extraction experiment ran until the silicon concentration was less than 0.5 mg L^−1^ and became constant. All samples were prepared in triplicate, and blank experiments (only soils) without biochar were also prepared. The column diagram can be drawn using average values and standard error in triplicate sample by Origin 8.5 software.

### Effect of FeCl_3_ on Biochar Adsorption of Silicic Acid

To investigate the effect of FeCl_3_ on biochar adsorption of silicic acid, an adsorption experiment was conducted in a 10 mL plastic centrifuge tube by mixing 50 mg of the biochar with 10 mL of 6 mg L^−1^ silicic acid with or without 2% FeCl_3_. The Si-rich RH biochars (RH300, RH500, and RH700) and RS biochars (RS300, RS500, and RS700) and the Si-deficient WB biochars (WB300, WB500, and WB700) and OP biochars (OP300, OP500, and OP700) were all tested. The pH of 6 mg L^−1^ silicic acid was adjusted to a suitable value to produce a solution equilibrium pH of 5.0. The tubes were shaken at 120 rpm for 3 d, 5 d, and 7 d, for biochars produced at pyrolytic temperature of 700, 500, and 300 °C, respectively. The solution was centrifuged at 3000 rpm for 5 min, the concentration of dissolved silicon was determined by silicon-molybdenum blue colorimetry, and the pH of the supernatant solution was also measured simultaneously. All samples were prepared in duplicate, and blank experiments without biochar were also prepared, and the statistical results can be acquired by using the average and standard deviation of duplicate sample. The surface transformation and distribution of elements on the biochar before and after adsorption was determined by SEM-EDS.

## Electronic supplementary material

Supporting Information
