# Supplementary material for: Biochar Impacts on Soil Silicon Dissolution Kinetics and their Interaction Mechanisms
Source: Sci Rep. 2018 May 23;8:8040. doi: 10.1038/s41598-018-26396-3 (PMC5966406; doi:10.1038/s41598-018-26396-3)
Supplement: Supplementary file 1 — Supporting Information [file 41598_2018_26396_MOESM1_ESM.pdf]

Supporting Information for *Scientific Reports*

Biochar Impacts on Soil Silicon Dissolution Kinetics and their Interaction Mechanisms

Yaofeng Wang,[†,‡] Xin Xiao, [†,‡] and Baoliang Chen,[*,†,‡]

[†]Department of Environmental Science, Zhejiang University, Hangzhou, Zhejiang 310058, China

[‡]Zhejiang Provincial Key Laboratory of Organic Pollution Process and Control, Hangzhou, Zhejiang 310058, China

Supporting Information consists of 7 pages, including this one.

There are 4 Figures.

Contents:

Page S2. Preparation of Si-rich and Si-deficient biochars.

Page S2. Biochar and soil structural characteristics

Page S4. **Figure S1.** The FTIR of RS700 and RH700 before and after washing (after washing indicated with “w-”).

Page S5. **Figure S2.** TOC content of the biochars before and after washing. The samples include the Si-rich biochars (RH300-RH700, RS300-RS700) and the Si-deficient biochars (WB300-WB700, OP300-OP700). The numbers represent the carbonization temperature.

Page S6. **Figure S3.** The efficiency of silicon dissolution for biochars. The samples include the Si-rich biochars (RH300-RH700, RS300-RS700) and the Si-deficient biochars (WB300-WB700, OP300-OP700). The numbers represent the carbonization temperature.

Page S7. **Figure S4.** Cumulative amount of silicon dissolved from the biochars alone. The solid-to-liquid ratio is 50 mg biochar 2000 mg soil/50 mL.

Preparation of Si-rich and Si-deficient biochars

RH and RS were obtained from a farm in the western Zijingang campus of Zhejiang University, China; WB was acquired from a farm in a wood factory, Huzhou, Zhejiang, China; and OP was collected from the fruit store at Zhejiang University. After air-drying for 5 days and oven-drying at 70 °C overnight, these materials were ground in a grinder and subsequently passed through a 100-mesh sieve. The biochar was prepared by pyrolyzing RH, RS, WB, and OP under oxygen-limited conditions at various temperatures. RH, RS, WB, or OP powder was tightly packed in a 100 mL ceramic pot that was subsequently placed in a chamber electric furnace (SSXF-4-10, Shanghai Haoyue Instrument and Equipment CO., LTD, China). The furnace was programmed with a heating rate of 5 °C min^{-1}, held for 6 h at a stable temperature (i.e., 300 °C, 500 °C, and 700 °C), and then allowed to cooldown to room temperature naturally. For the Si-rich materials (rice husk, rice straw), the residue materials were designated RH300, RH500, and RH700, RS300, RS500, and RS700, respectively, and for the Si-deficient materials (wood sawdust, orange peel), the residue materials were designated WB300, WB500, and WB700, OP300, OP500, and OP700, respectively, where the suffix number indicates the carbonization temperature. The biochar samples were then passed through a 100-mesh sieve for subsequent experiments.

Biochar and soil structural characteristics

The soil available silicon was measured by placing 10g soil samples 250mL plastic bottles with 100ml 0.025mol L^{-1} citric acid and determined by silicon-molybdenum blue colorimetry (Chinese national agricultural standards NY/T 1121.15-2006). Amorphous iron oxides in soil were determined by hydroxylamine extraction spectrophotometry and the soil total silicon was determined by using a sodium hydroxide melt and silicon-molybdenum blue colorimetry determination. The organic carbon content of the biochars were measured using a TOC-V carbon analysis instrument (SSM-5000A, Shimadzu, Japan). The ash content was determined by heating the biochar samples at 800 °C under an air atmosphere for 4 h. The surface area (SA) of the biochar was determined by N_2 gas adsorption-desorption using a NOVA-2000E surface area analyzer. The surface area (SA) was measured by a multipoint BET-N_2 analysis of the adsorption data points with relative pressures 0.05-0.3. The biochar samples before and after water washing were characterized using Fourier transform infrared spectroscopy (FTIR), and scanning electron microscopy with energy dispersive

X-ray spectrometry (SEM-EDX). For the FTIR analysis, the samples were mixed with potassium bromide at 1/100 (w/w) and then pressed into lamellar form. FTIR (Nicolet 6700 spectrometer) spectra were recorded in the 400−4000 cm^{-1} region, and 64 interferograms were collected with a resolution of 1 cm^{-1} for each FTIR spectrum. Energy-dispersive X-ray spectroscopy (EDX) analyses were performed on an SU-8010 scanning electron microscope (SEM, HITACHI, Japan) with an acceleration voltage of 5.0 kV. Biochar samples were spread on a carbon tape and then polished with a thin platinum layer to enhance the electrical conductivity of the surface and improve the image quality.

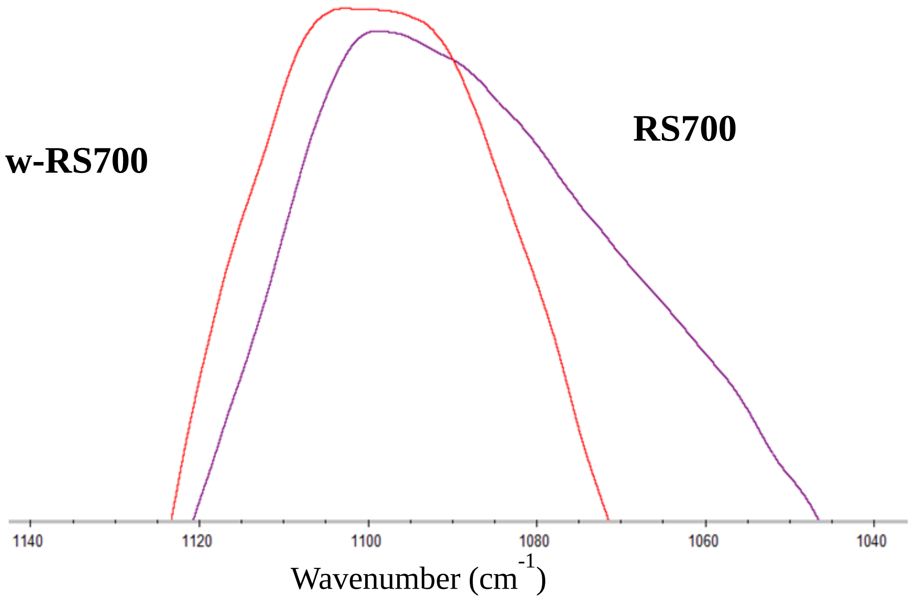

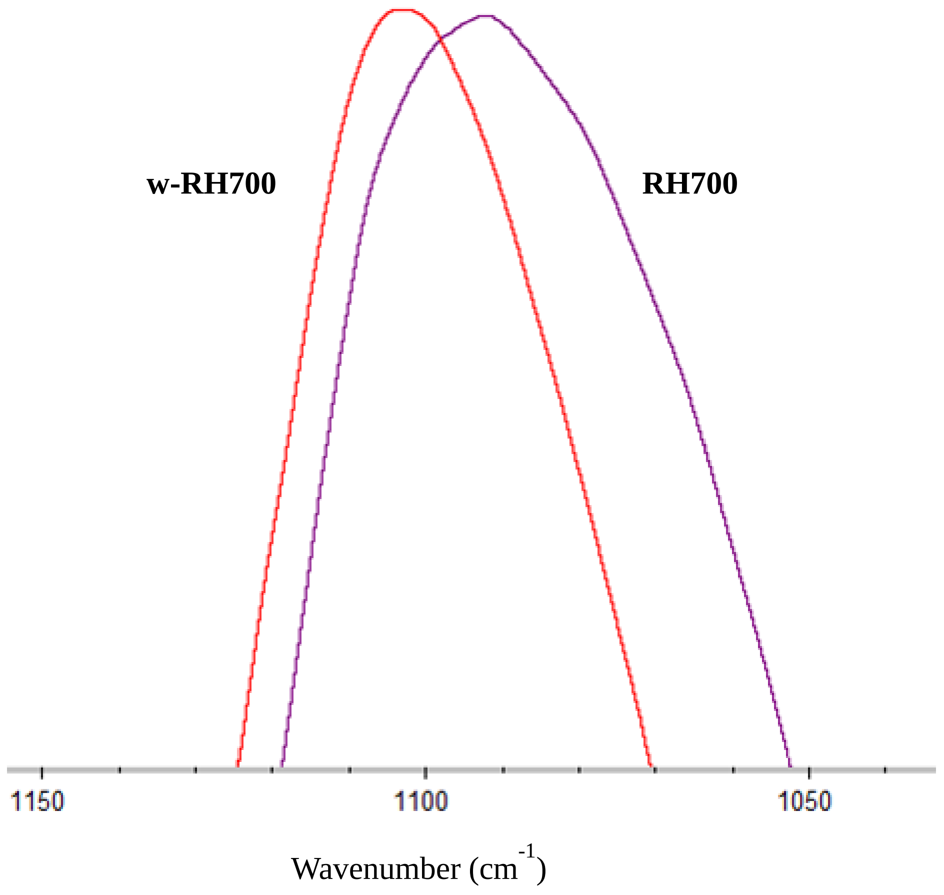

Figure S1. The FTIR of RS700 and RH700 before and after washing (after washing indicated with "w-").

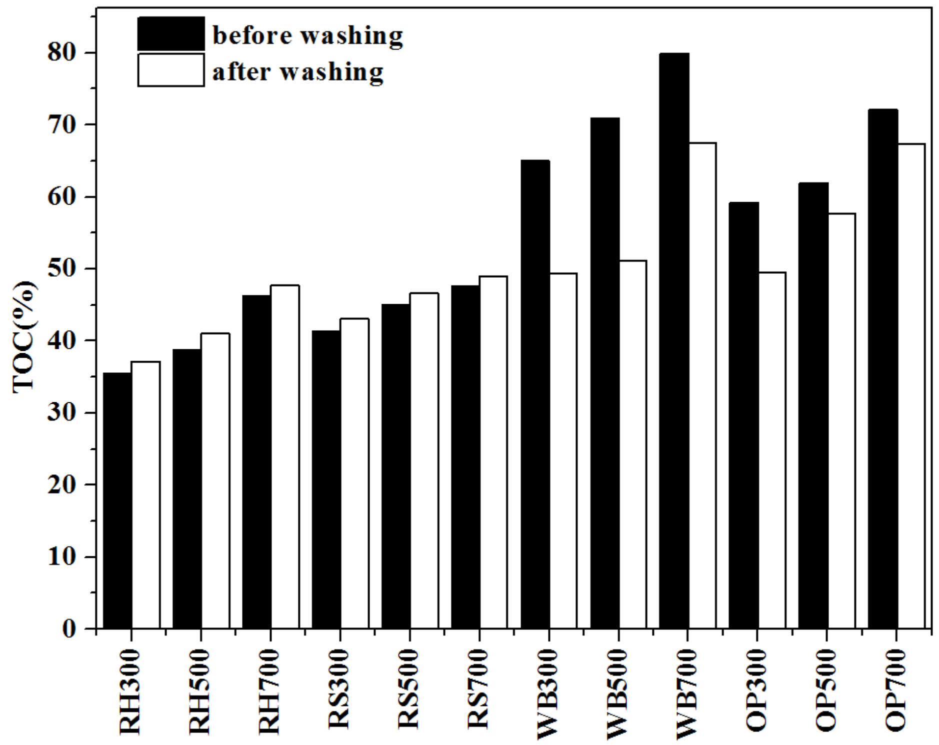

Figure S2. TOC content of the biochars before and after washing. The samples include the Si-rich biochars (RH300-RH700, RS300-RS700) and the Si-deficient biochars (WB300-WB700, OP300-OP700). The numbers represent the carbonization temperature.

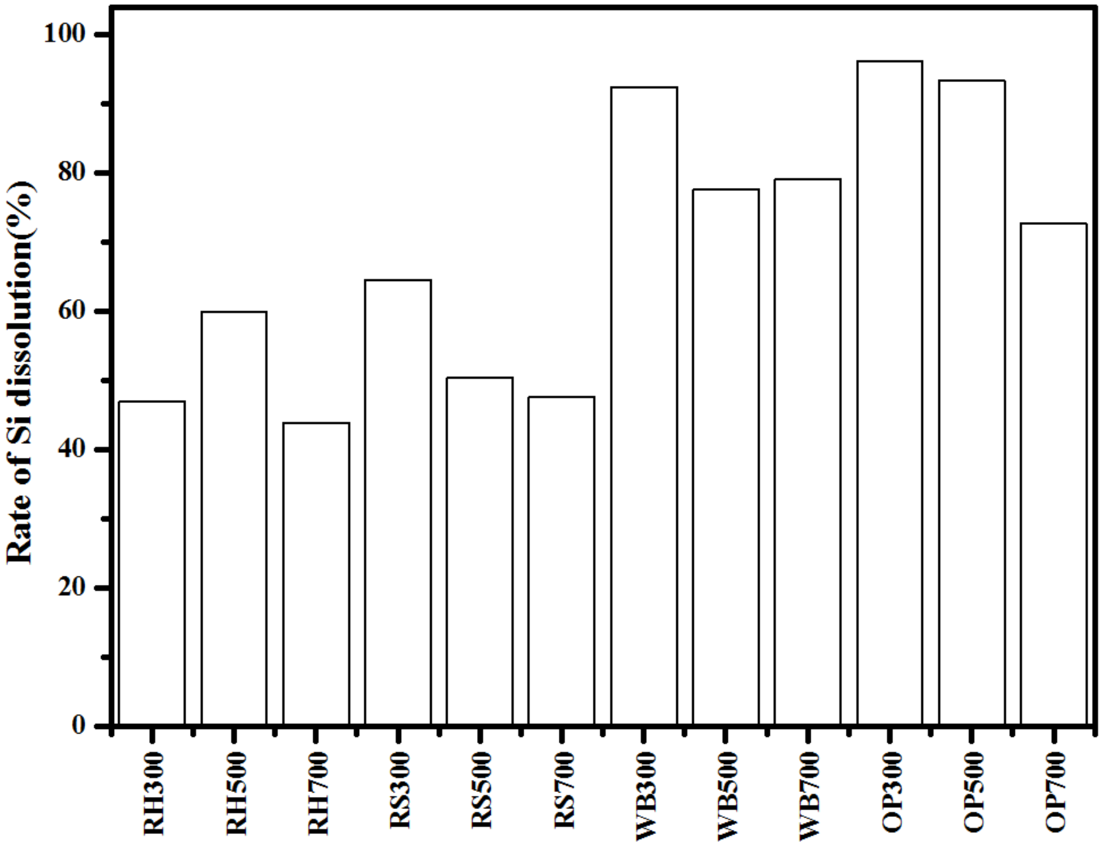

Figure S3. The efficiency of silicon dissolution for biochars. The samples include the Si-rich biochars (RH300-RH700, RS300-RS700) and the Si-deficient biochars (WB300-WB700, OP300-OP700). The numbers represent the carbonization temperature.

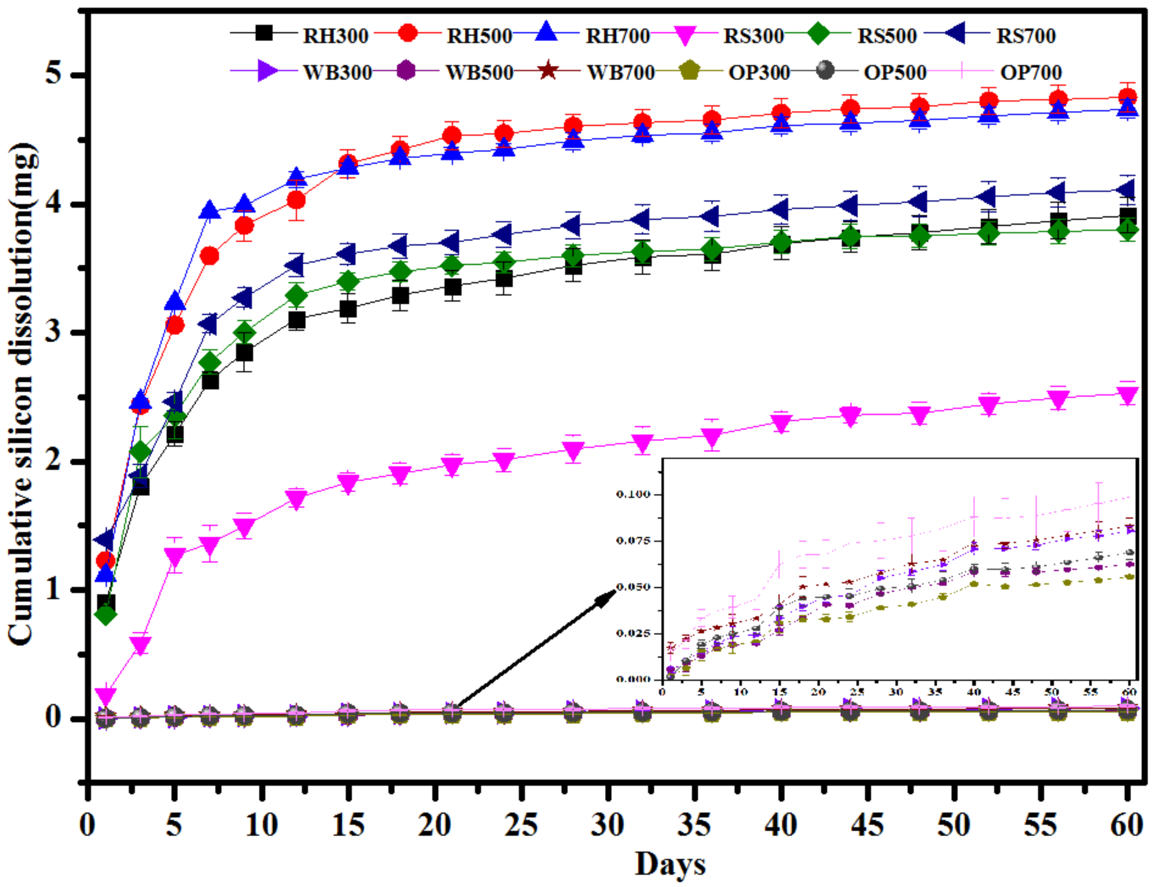

Figure S4. Cumulative amount of silicon dissolved from the biochars alone. The solid-to-liquid ratio is 50 mg biochar /50 mL Milli-Q water. The numbers represent the carbonization temperature.
